# ALK-positive diffuse large B-cell lymphoma: report of four cases and review of the literature

**DOI:** 10.1186/1756-8722-2-11

**Published:** 2009-02-27

**Authors:** Brady Beltran, Jorge Castillo, Renzo Salas, Pilar Quiñones, Domingo Morales, Fernando Hurtado, Luis Riva, Eric Winer

**Affiliations:** 1 Department of Oncology and Radiotherapy, Edgardo Rebagliati Martins Hospital, Lima, Peru; 2 Division of Hematology and Oncology, The Miriam Hospital, Brown University Warren Alpert Medical School, Providence, RI, USA; 3 Department of Pathology, Edgardo Rebaglati Martins Hospital, Lima, Peru

## Abstract

**Background:**

Anaplastic lymphoma kinase-positive diffuse large B-cell lymphoma (ALK-DLBCL) is a rare lymphoma with several clinicopathological differences from ALK-positive anaplastic large cell lymphoma (ALCL). The latest WHO classification of lymphomas recognizes ALK-DLBCL as a separate entity.

**Methods:**

A comprehensive comparison was made between the clinical and pathological features of the 4 cases reported and those found in an extensive literature search using MEDLINE through December 2008.

**Results:**

In our series, three cases were adults and one was pediatric. Two cases had primary extranodal disease (multifocal bone and right nasal fossa). Stages were I (n = 1), II (n = 1), III (n = 1) and IV (n = 1). Two cases had increased LDH levels and three reported B symptoms. IPI scores were 0 (n = 1), 2 (n = 2) and 3 (n = 1). All cases exhibited plasmablastic morphology. By immunohistochemistry, cases were positive for cytoplasmic ALK, MUM1, CD45, and EMA; they marked negative for CD3, CD30 and CD20. Studies for EBV and HHV-8 were negative. The survival for the patients with stage I, II, III and IV were 13, 62, 72 and 11 months, respectively.

**Conclusion:**

ALK-DLBCL is a distinct variant of DLBCL with plasmacytic differentiation, which is characterized by a bimodal age incidence curve, primarily nodal involvement, plasmablastic morphology, lack of expression of CD20, aggressive behavior and poor response to standard therapies, although some cases can have prolonged survival as the cases reported in this study. ALK-DLBCL does not seem associated to immunosuppression or the presence of EBV or HHV8. Further prospective studies are needed to optimize therapies for this entity.

## Background

DLBCL is the most common histological variant of NHL. It encompasses multiple subtypes and has heterogeneous clinical and pathological features. In 1997, Delsol and colleagues reported seven cases of a distinct variant of DLBCL expressing rearrangements of the ALK gene [1]. The plasmablastic appearance and CD20-negativity of ALK-DLBCL makes this entity a potentially diagnostic challenge with a broad differential diagnosis. Clinically, ALK-DLBCL shows very aggressive behavior, high relapse rate and lack of response to standard regimens.

Although in the initial report by Delsol and colleagues the classic ALK gene rearrangement observed in ALCL could not be shown [1], modern techniques have been able to prove recurrent chromosomal abnormalities in ALK-DLBCL. The most commonly observed cytogenetic abnormality is t(2;17)(p23;q23) or clathrin/ALK [2-10]. The classic ALCL-related t(2;5)(p23;q35) or nucleophosmin/ALK has also been described [11-13]. Other rare cytogenetic abnormalities have been reported [14,15].

The main objective of this study was to describe the clinicopathological characteristics of four additional cases of ALK-DLBCL and compare them with those of 46 literature-reported cases.

## Materials and methods

Four cases of ALK-DLBCL were identified from the Hematology and Medical Oncology consultation files at the Edgardo Rebagliati Martins Hospital in Lima, Peru between January 1, 1997 and June 30, 2008. Clinical and laboratory information for each of the four patients was obtained through physician interview and medical chart review, after approval of this study by the IRB. Routine hematoxylin and eosin-stained sections were prepared from formalin-fixed and/or B5-fixed paraffin blocks. Immunohistochemical analysis included a broad panel of antibodies against ALK1 (Dako, Carpinteria, CA; dilution 1:50), CD45 (Dako; dilution 1:400), CD4 (Novocastra, Newcastle upon Tyne, UK; dilution 1:20), CD56 (Sanbio, Uden, The Netherlands; 1:200), CD20 (Dako; dilution 1:100), CD79a (Dako; dilution 1:25) and light chains of immunoglobulin. The samples were also stained for CD30 (Novocastra; dilution 1:100) and EMA (Dako; dilution 1:50), which are usually expressed by ALCL cells.

Immunohistochemical studies for Epstein Barr virus (EBV) and human herpesvirus 8 (HHV-8) were performed at the Department of Pathology of the Rhode Island Hospital in Providence, RI. EBV clone was CS1-4 (Dako; dilution 1:500) obtained through heat retrieval pretreatment with Target Retrieval solution (Dako) for 25 minutes. HHV8 clone was 13B10 (Vector Laboratories, Burlingame, CA; dilution 1:50) obtained through heat retrieval pretreatment with Target Retrieval solution (Dako) for 25 minutes. Cytogenetic studies by FISH looking for ALK gene rearrangement were performed at the Department of Cytogenetics of the Tufts Medical Center in Boston, MA. The immunohistochemical analysis for HHV-8 and cytogenetic studies were performed in only two of the present cases. Further studies could not be attempted on the other two cases due to lack of available remaining specimen.

For the review, we performed a literature search using Pubmed/MEDLINE looking for articles reporting clinicopathological data in patients with ALK-DLBCL through December 2008. Eighteen articles were considered for this review. Data were gathered on age, sex, pattern of ALK expression, ALK gene rearrangement variety, expression of CD30, CD45, plasma cell, B-cell, T-cell and NK-cell markers, EMA and light chain, heavy chain gene and T-cell receptor gene rearrangements, presence of EBV, site of primary disease, clinical stage, LDH levels, IPI score, therapy at presentation and at relapse, outcome, survival in months and cause of death. Survival analyses were attempted using Kaplan-Meier estimates for age, sex, T-cell marker expression, primary site of presentation, clinical stage, LDH levels and IPI score. All reported p-values are two-sided.

## Results

### Case Reports

A summary of the clinical features of the four patients is provided in Table 1.

**Table 1:** Clinical characteristics of the reported cases

**Case**	**Age**	**Sex**	**Primary site**	**Bone marrow involvement**	**Stage**	**IPI**	**Therapy**	**Survival (Months)**	**Outcome**
1	27	M	Bone	Yes	IVB	3	HyperCVAD	11	Alive, with disease

2	41	F	Nasal fossa	No	IA	0	Radiotherapy	13	Alive, NED

3	13	F	Cervical LN	No	IIB	2	LNH96-2002	62	Alive, NED

4	70	M	Cervical LN	No	IIIB	3	CHOP	72	Alive, NED

IPI – International Prognostic Index.

NED – no evidence of disease.

LN – lymph node.

HyperCVAD – hyperfractionated cyclophosphamide, vincristine, doxorubicin and dexamethasone alternating with cytarabine and methotrexate.

CHOP – cyclophosphamide, doxorubicin, vincristine, prednisone.

#### Case 1

A 27-year-old male patient presented with multifocal bone lesions detected with bone scintigraphy. Patient also reported the presence of B symptoms. LDH levels were elevated. Serum protein electrophoresis (SPEP) did not show a monoclonal spike. A computed tomography (CT) scan of the thorax and abdomen showed no mass lesions or additional lymphadenopathy. An incisional biopsy of bone was performed, which showed a diffuse lymphoma of plasmablastic appearance. A staging bone marrow aspiration and biopsy was positive for involvement by lymphoma. Patient was staged as IVB and underwent six cycles of EPOCH (cyclophosphamide, vincristine, doxorubicin, etoposide and prednisone) with persistent bone marrow infiltration at the end of the initial therapy. He is currently receiving hyperCVAD (hyperfractionated cyclophosphamide, vincristine, doxorubicin and dexamethasone alternating with cytarabine and methotrexate). At 11 months, he was alive with persistent disease.

#### Case 2

A 41-year-old male patient presented with history of nasal obstruction for one month. He was otherwise asymptomatic with an excellent performance status and had no significant past medical history. Hematologic, basic metabolic, liver function studies and LDH levels were within normal limits. SPEP did not show monoclonal spike. CT scan of the head, neck, chest, abdomen and pelvis revealed only a mass in the right nasal fossa. Biopsy of tumor was performed revealing a tumor with plasmablastic morphology. Staging bone marrow was negative. Due to an initial diagnosis of solitary plasmacytoma, patient received involved field radiation therapy. At 13 months, he was alive and free of disease.

#### Case 3

A 13-year-old female patient presented with a rapidly enlarging left neck mass and B symptoms. Physical examination and radiological studies showed axillary and mediastinal lymph nodes and costal bone involvement. A biopsy of the cervical mass was performed and revealed an aggressive lymphoma with plasmablastic features. Bone marrow biopsy was negative for lymphoma. SPEP was not performed. She received the regimen LNH96-2002, which is based on induction with vincristine, prednisone, cyclophosphamide, daunorubicin, L-asparaginase and methotrexate; followed by consolidation based on cyclophosphamide, cytarabine, methotrexate then intensification with vincristine and doxorubicin and maintenance based on methotrexate and mercaptopurine. She had a complete response to the induction phase and then received consolidation and maintenance. She has 62 months alive and free from recurrence.

#### Case 4

A 70-year-old male patient presented with cervical, axillary and inguinal lymphadenopathy without B symptoms. Bone marrow was not involved. He had a performance status of 2. Cervical lymph node biopsy was done showing a diffuse lymphoma with plasmablastic appearance. LDH levels were within normal limits. SPEP was not performed. Patient was considered stage IIIB. IPI score was 3 out of 5. He received CHOP-21 regimen for six cycles and achieved a complete response. He is alive with 72 months free from recurrence.

#### Pathological aspects of the reported cases

**Figure 1:**
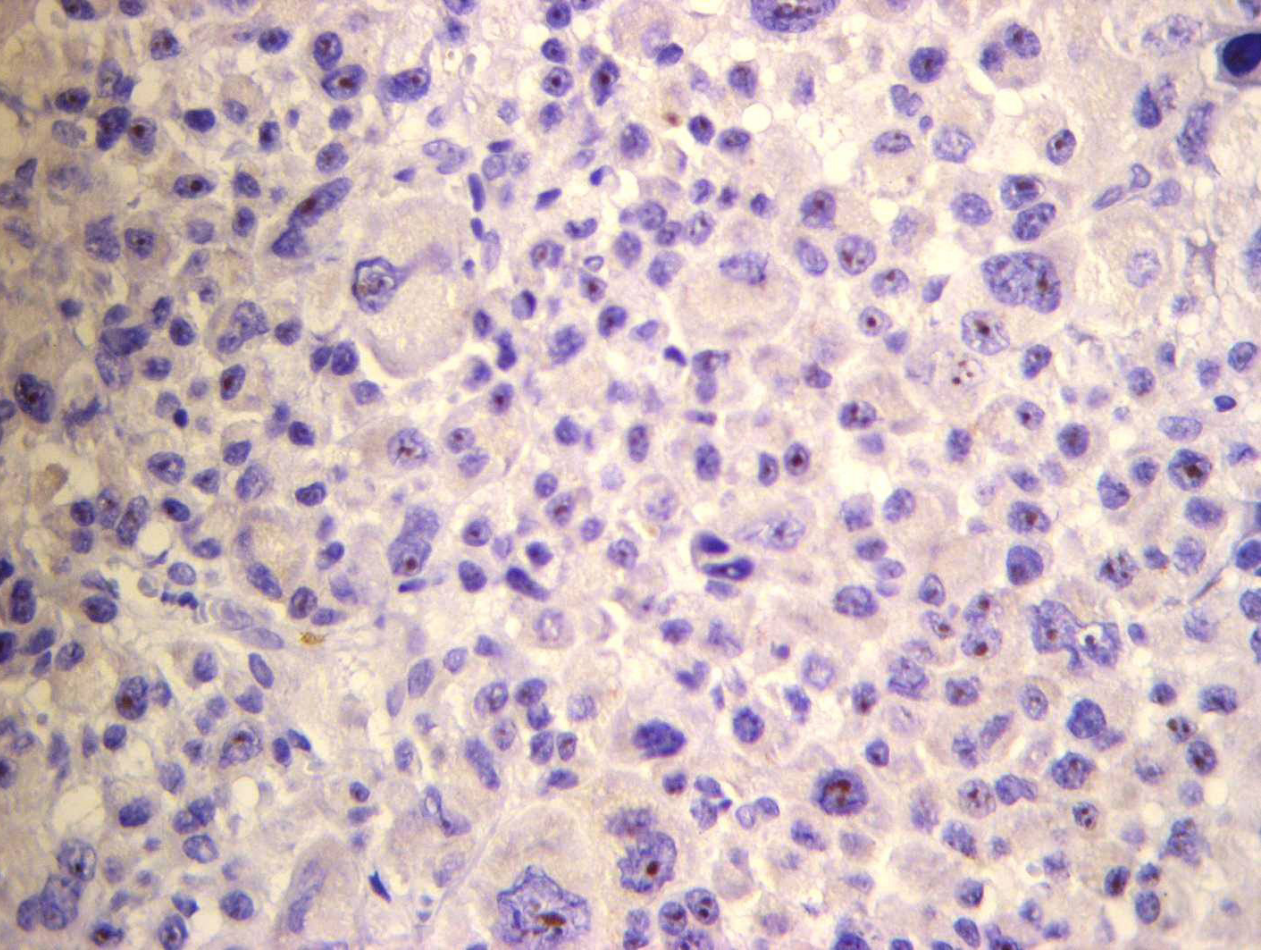
**Negative CD20 expression in ALK-DLBCL**.

**Figure 2:**
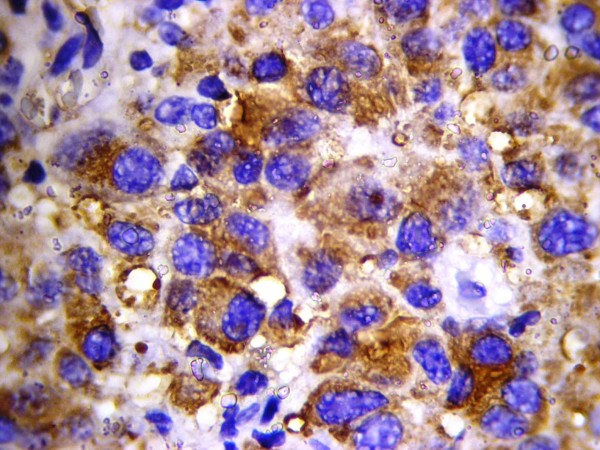
**MUM1 expression in ALK-DLBCL**.

**Figure 3:**
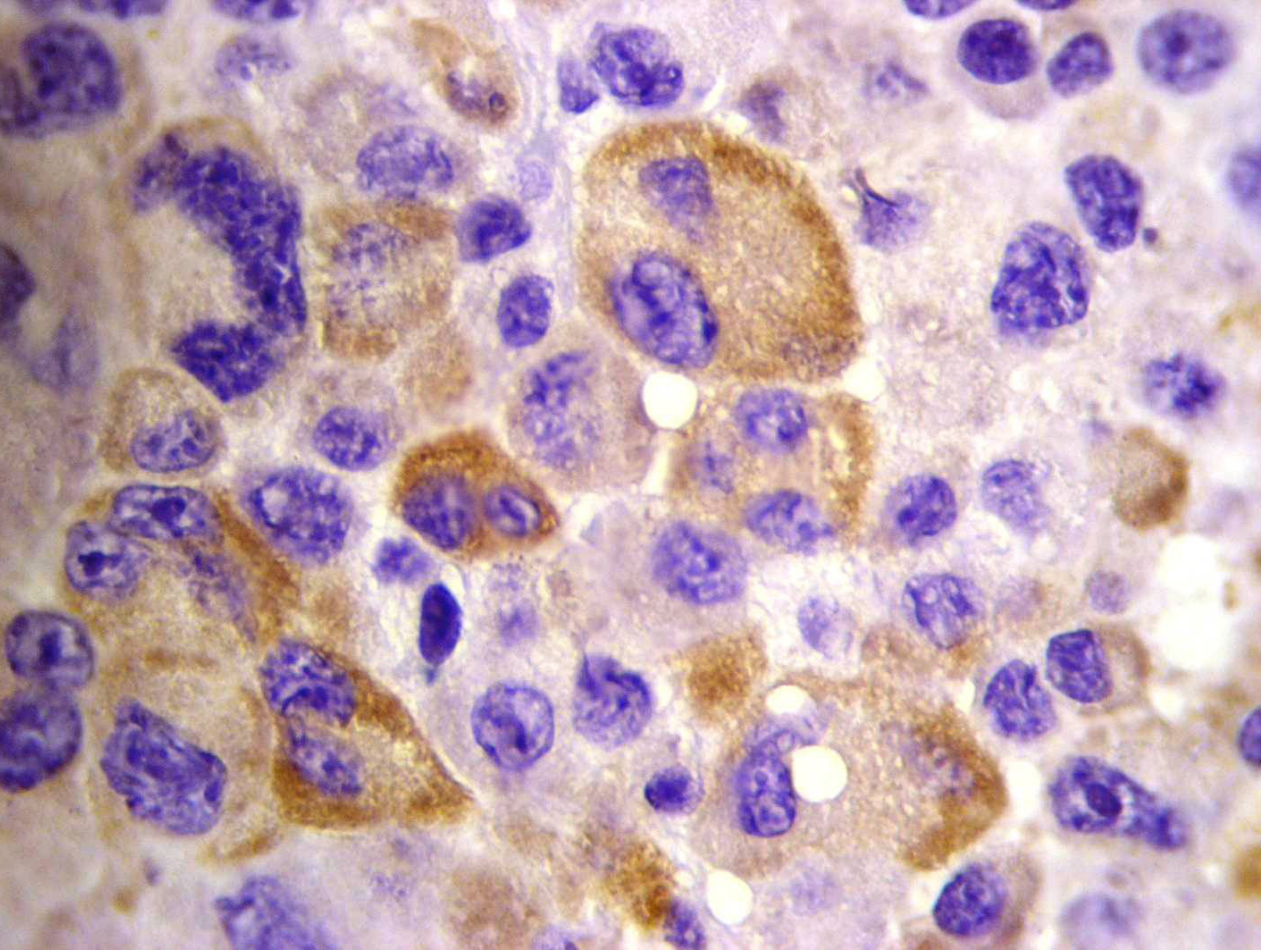
**EMA expression in ALK-DLBCL**.

**Figure 4:**
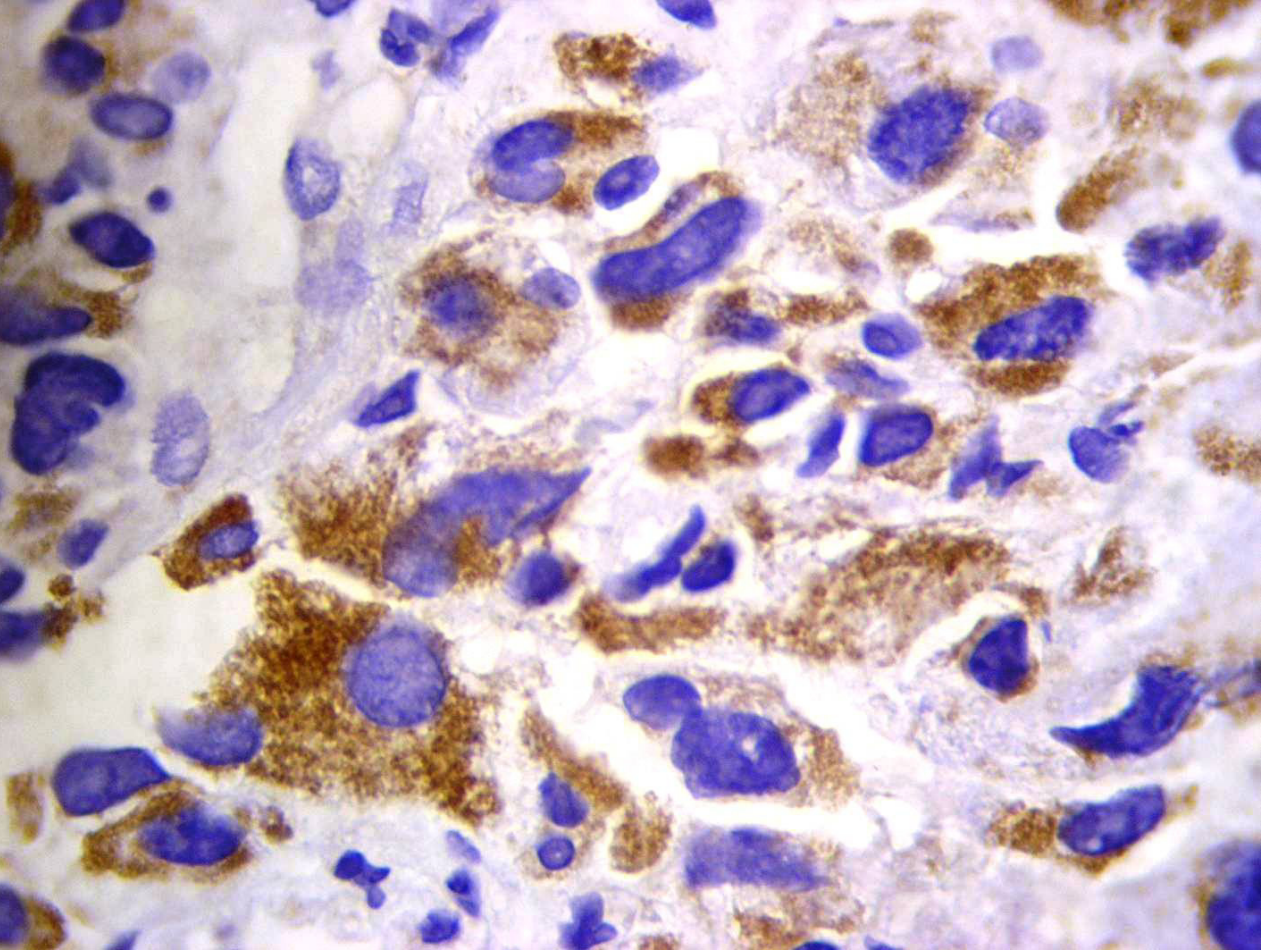
**Granular cytoplasmic ALK expression in ALK-DLBCL**.

All four cases showed plasmablastic morphologic features with effacement of the normal architecture by sheets of tumor cells. The neoplastic cells in all cases were large with round, regular, with centrally located nuclei, dispersed chromatin, single central, prominent nucleolus, and moderate eosinophilic or amphophilic cytoplasm. Table 2 provides a summary of the immunohistochemical characteristics in the four reported cases. All tested cases were positive for CD45, MUM1 (Figure 1), and EMA (Figure 2), and were negative for CD4, CD20 (Figure 3) and CD30. All cases were positive for ALK in a granular cytoplasmic distribution (Figure 4), which has been described in clathrin/ALK-associated cases. FISH by standard methods was unsuccessful as the examined pathological samples were decalcified causing excessive background autofluorescence.

**Table 2:** Morphology and immunohistochemical characteristics of the reported cases

**Case**	**Morphology**	**ALK**	**CD45**	**CD20**	**CD79a**	**CD4**	**CD56**	**MUM1**	**CD30**	**EMA**	**Lambda**	**EBV**	**HHV8**
1	Plasmablastic	+	+	-	-	-	-	+	-	ND	+	-	ND

2	Plasmablastic	+	+	-	+	-	-	+	-	+	+	-	-

3	Plasmablastic	+	+	-	-	-	-	+	-	+	+	-	-

4	Plasmablastic	+	+	-	-	-	-	+	-	+	-	-	ND

ALK – anaplastic lymphoma kinase.

EMA – epithelial membrane antigen.

EBV – Epstein Barr virus.

HHV8 – human herpesvirus 8.

ND – not done.

## Discussion and review of the literature

### Pathological aspects

### Morphological features

ALK-DLBCL is an entity with immunoblastic or plasmablastic microscopical appearance with round nuclei, prominent single central nucleoli, and moderate amounts of variably eosinophilic cytoplasm.

DLBCL with plasmablastic features and terminal B-cell differentiation represents a heterogeneous spectrum of distinct entities [16]. Differential diagnosis of ALK-DLBCL should include lymphoblastic lymphoma, anaplastic variants of DLBCL, plasmablastic lymphoma (PBL), primary effusion lymphoma (PEL), solid variants of PEL and plasmablastic myeloma.

It is important to note that few cases of ALK-DLBCL were treated initially as ALCL due to morphological appearance, CD20-negativity and presence of ALK-positive staining [5,7]. ALK-positive ALCL, although a T-cell lymphoma, should be considered in the differential diagnosis of ALK-DLBCL given its good prognosis [17].

### Immunohistochemistry (see Table 3)

**Table 3:** Immunohistochemical and molecular features of 50 cases of ALK-DLBCL reported in the literature

	**Number studied**	**Number positive/weak**	**%**
**Immunohistochemistry**			
ALK	50	50	100
Cytoplasmic		43	86
Nuclear		6	12
Other		1	2
VS38c/CD138/MUM1	39	39	100
EMA	38	37	97
CD45	27	19/2	78
CD4	40	11/5	40
CD57	24	3/5	33
Perforin	24	2	8
CD20	44	4/1	11
CD79a	44	6/2	18
CD30	45	5	11
EBV	17	0	0
HHV8	2	0	0

**Molecular studies**			
ALK gene rearrangement	24	24	100
Clathrin/ALK		18	75
Nucleophosmin/ALK		4	16
Other rearrangements		2	8
IgH gene rearrangement	20	17	85
TCR gene rearrangement	4	1	25
EBER CISH	12	0	0

ALK – anaplastic lymphoma kinase

EMA – epithelial membrane antigen

EBV – Epstein Barr virus

HHV8 – human herpesvirus 8

IgH – immunoglobulin heavy chain

TCR – T-cell receptor

EBER – EBV-encoded RNA

CISH – chromogenic in situ hybridization

The most commonly observed ALK staining pattern was cytoplasmic and granular, caused by clathrin-ALK fusion. This pattern is explained by the function of clathrin, which is present in coated vesicles necessary for at least 50% of the endocytic activity of the cell [18,19]. In contrast, the NPM-ALK fusion protein seen in ALCL has a characteristic nuclear and cytoplasmic sub-cellular localization pattern, which was found in a few cases. The gene NPM1, which codes for nucleophosmin, is frequently overexpressed and rearranged in human cancer and has proto-oncogenic and tumor suppressor features [20].

ALK-DLBCL presents 100% positivity for plasmacytic differentiation markers like CD138, VS38c and MUM1; EMA was expressed in 97% of the cases. B-cell related antigens such as CD20 and CD79a were rarely expressed in ALK-DLBCL (11% and 18%, respectively). These observations support the inference that ALK-DLBCL is derived from post-germinal B-cell lymphocytes that have undergone class switching and plasmacytic differentiation. Furthermore, expression of monotypic cytoplasmic light chain occurred in 85% of all cases. Based on these findings, ALK-DLBCL falls into the category of non-GC DLBCL. Patients with DLBCL of non-GC molecular or immunhistochemical profile have worse clinical outcomes than their counterparts of GC-like origin [21-23]. Higher intensity regimens or agents used in therapy of plasma cell myeloma should undergo prospective studies in this population, which is unlikely to have higher benefits from current standard therapies (i.e. CHOP).

CD45 was expressed variably positive in 70% of cases, T-cell markers like CD4 was found in 40% of cases and NK markers like CD57 was positive in 33% of cases. T-cell marker expression did not play a role in survival (p = 0.37). The reason for aberrant T-cell and/or NK-cell markers expression is unknown; however, unusual T-cell markers expression has been seen in other B-cell lymphoproliferative conditions such as CLL, HCL and MCL [24]. CD56 has also been found expressed in B-cell lymphomas such as DLBCL and FL [25]. The clinical impact of aberrant T-cell or NK-cell markers in B-cell lymphoproliferative disorders is unknown but deserves attention for potential diagnostic, prognostic and/or therapeutic approaches.

The four cases reported in the present study were negative for the presence of EBV using LMP-1. From the literature, 12 cases were negative using EBER chromogenic in situ hybridization (CISH), which is more sensitive than LMP-1. Hence, ALK-DLBCL does not seem to be associated to EBV. In contrast, EBV has been associated with other DLBCL with plasmacytic differentiation such as plasmablastic lymphoma in HIV-infected patients [26]. The presence of HHV-8 was evaluated in two cases of our series and was negative in both. HHV-8 is involved in the pathogenesis of other entities with terminal B-cell differentiation such as classic and solid variants of PEL [27]. No virus has been associated to the development of ALK-DLBCL thus far.

### Molecular studies (see Table 3)

As mentioned above, the most frequent ALK gene rearrangement was clathrin-ALK in 75% of cases; however 17% corresponded to NPM-ALK fusion. ALK gene is located on chromosome 2p23 and encodes a tyrosine kinase receptor belonging to the insulin receptor superfamily, which is normally silent in lymphoid cells [28] and it could be translocated to either the clathrin gene locus located on chromosome 17q23 or to the NPM1 gene located on chromosome 5q35, constituting the clathrin-ALK and NPM-ALK fusion products, respectively. In few cases, the actual ALK gene rearrangement could not be demonstrated or was not reported [29-32].

All ALK fusion proteins share two essential characteristics: 1) presence of an N-terminal partner protein, a gene promoter which controls aberrant transcription of ALK chimeric mRNA and the expression of its encoded fusion protein, and 2) presence of an oligomerization domain in the sequence of the ALK fusion partner protein which mediate constitutive self association of the ALK fusion causing constant ALK domain activation. Oncogenesis occurs from ensuing dimerization leading to constitutive activation of ALK tyrosine kinase activity. Stachurski and colleagues [15] described a novel mechanism of ALK activation by means a cryptic 3'ALK gene insertion into chromosome 4q22-24. The role of this anomaly in lymphomagenesis is unclear.

Immunoglobulin heavy chain gene rearrangements were detected by PCR analysis in 17 of 20 studied cases (85%). The previous finding, along with the expression of monotypic cytoplasmic immunoglobulin light chain, confirms the B-cell lineage of this disorder.

### Clinical aspects (see Table 4)

**Table 4:** Clinical features of 50 cases of ALK-DLBCL reported in the literature

	**N**	**%/range**
Age, years (n = 47)	38	9 – 72

Sex (n = 50)		

Male	38	76

Female	12	24

Site of involvement (n = 46)		
Exclusively nodal	24	52
Cervical	17	71
Other	7	29
Extranodal	22	48
Bone	8	36
Liver and spleen	4	18
Head and neck	3	14
Gastrointestinal tract	3	14
Other*	8	36

Clinical stage (n = 47)		
I – II	20	43
III – IV	27	57

Therapy (n = 41)		
Chemotherapy	34	83
Chemoradiotherapy	6	15
Radiotherapy	1	2

Relapsed cases	18	44

Salvage HSCT	8	20

Survival time, months (n = 36)	24	3 – 156

HSCT – hematopoietic stem cell transplantation

*Includes bone marrow, CNS, gonads and muscle

#### Age and sex distribution

**Figure 5:**
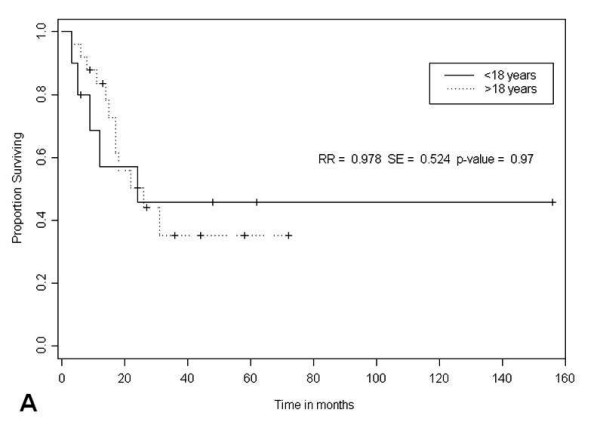
**Kaplan-Meier survival estimates according to age in 50 ALK-DLBCL cases from the literature**.

Forty-seven cases of ALK-DLBCL reported age of presentation. The average age of presentation was 38 years, ranging from 9 to 72 years of age. Despite the small amount of cases, we can already observe a bimodal age distribution. Eleven cases of ALK-DLBCL have been reported in pediatric population [2,5,7,8,12], accounting for 24% of the total number of cases. In patients younger than 18 years, the average age of presentation was 12.4 years and in adults it was 43.4 years. There was no difference in survival between pediatric and adult cases (p = 0.97; Figure 5), despite more intensive therapies in pediatric population.

In regards of sex distribution, the male to female ratio was 3:1; female cases accounted for 23% of the cases. In pediatric cases, the male to female ratio was 1.8:1 and in adults 4.3:1. There was no statistical difference between the overall survival of men compared to women (p = 0.45).

#### Sites of involvement

Data on primary sites of presentation were available in 46 cases. Twenty-four cases (52%) were exclusively nodal in origin. The most commonly affected areas were cervical and mediastinal. Few cases presented with generalized lymphadenopathy. The remaining cases (48%) had some extranodal component and from these, only 6 were exclusively extranodal. Most common extranodal sites of disease were bone, liver, spleen, gastrointestinal tract and the head and neck region.

ALK-DLBCL differs somewhat from other subtypes of DLBCL with plasmacytic differentiation. Plasmablastic lymphoma (PBL), a CD20-negative DLBCL associated to HIV and EBV coinfection, tends to present with extranodal involvement, usually in oral and gastrointestinal sites; nodal presentation in PBL has been reported in only 6% of the cases [26]. In a similar fashion, PEL, another CD20-negative DLBCL seen exclusively in association with HHV-8, tends to present in body cavities such as pleura and peritoneum [27]. Although nodal PEL has been described [33], available data on extracavitary or solid variants of PEL is very limited. In the survival analysis, there was no statistical difference between nodal and extranodal sites of involvement (p = 0.58).

#### Clinical stage and IPI score

**Figure 6:**
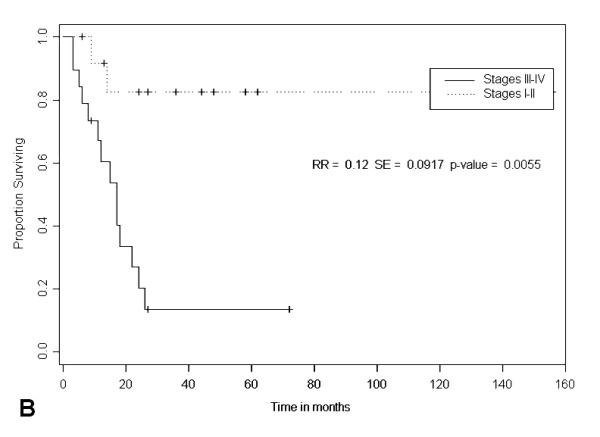
**Kaplan-Meier survival estimates according to clinical stage in 50 ALK-DLBCL cases from the literature**.

From 47 ALK-DLBCL cases of the literature, advanced stage (i.e. III and IV) was reported in 57% of the cases; the remainder 43% presented with stages I or II. As observed in other malignant lymphoproliferative disorders, clinical stage had a strong correlation with survival (p = 0.0055; Figure 6).

The IPI score has been accepted as the standard method for risk stratification in patients with DLBCL [34]. Unfortunately, only 8 of the 50 reported cases (17%) had available data on IPI scores, including the 4 cases reported in this study. Furthermore, the gathered data did not allow the authors to calculate IPI scores as serum LDH levels and performance status were seldom reported. Survival analyses using IPI scores or LDH levels were not performed.

#### Therapy and relapses

Data on therapy was available in 41 ALK-DLBCL cases. Of these 32 cases (83%) received combination chemotherapy, 6 cases (15%) received chemoradiotherapy and one case (2%) received radiotherapy. Only one case [6] received immunotherapy with rituximab despite the CD20-negative nature of ALK-DLBCL; this patient died of lymphoma 6 months after diagnosis. From the 34 cases treated with chemotherapy, 12 cases (38%) were treated with CHOP and the remaining 20 cases (62%) were treated with more intensive regimens. From the 12 cases treated with CHOP, 6 cases (50%) needed more therapy due to relapsing disease and 4 cases (33%) died of progressive lymphoma.

In the 11 ALK-DLBCL pediatric cases, all regimens used were highly intensive (i.e. BFM90 [35], LMB89 [36], LMB96 [37], POG8719 [38]). Most of these regimens are used successfully to treat children with lymphoblastic, Burkitt and aggressive B-cell lymphomas. Of note, some of the patients were enrolled in randomized clinical trials that are still undergoing recruitment (i.e. ALCL99 [39]). In contrast with the reported efficacy of these regimens in other types of aggressive NHL, the success in ALK-DLBCL was rather moderate with 6 patients (55%) alive at the time of report.

In total, 18 ALK-DLBCL cases (44%) experienced refractory or relapsing disease. Most salvage regimens were based on platinum-containing compounds (i.e. ESHAP, DHAP, ICE). Hematopoietic stem cell transplantation (HSCT) was performed in 8 of the refractory or relapsing cases (44%) [1,5,7,10,11,14,30]. Four patients received autologous HSCT, one patient underwent allogeneic HSCT and 3 patients were treated with non-specified HSCT. All but one of the cases died after transplantation; the range of survival in transplanted cases was between 3 and 44 months after diagnosis. The case treated with allogeneic HSCT died of thrombotic thrombocytopenic purpura (TTP) 7 months after transplantation [30].

From the 50 cases of the literature, the authors could observe that standard CHOP regimen seems inadequate to treat ALK-DLBCL given evidence of progressive disease and multiple recurrences. The lack of expression of CD20 antigen in most cases of ALK-DLBCL makes the therapeutic role of rituximab rather unclear. Nonetheless, rituximab should be used in the few CD20-expressing ALK-DLBCL cases [32].

#### Outcome and survival

ALK-DLBCL was fatal in 56% of the cases. The most common cause of death was progressive lymphoma, observed in 90% of the reported cases. Other causes of death included TTP and infectious complications. The average survival for the 36 cases in which survival times were reported was 24 months. Multiple clinical factors such as age, sex and nodal primary sites do not seem to correlate with survival in ALK-DLBCL. The strongest factor associated to survival in ALK-DLBCL cases from the literature was clinical stage at presentation; patients with advanced stages had a median survival of 18 months while patients with earlier stages have not reached their median survival (Figure 6).

## Conclusion

ALK-DLBCL is a distinct subtype of DLBCL with plasmacytic differentiation that affects pediatric and adult patients. It has characteristic genetic abnormalities and corresponding specific ALK-staining patterns with a prognosis that depends largely on clinical stage. The clinical course of ALK-DLBCL is aggressive with primary refractory disease and high relapse rates. The classical CHOP regimen appears insufficient to treat this condition and newer, more intensive therapies are needed. Given its CD20-negativity, the role of rituximab in the treatment of ALK-DLBCL is unclear. It would be of interest to try agents borrowed from plasma cell myeloma regimens or agents active in novel pathways in combination with chemotherapy given ALK-DLBCL plasmacytic nature. Despite this aggressiveness, some cases, even in advanced stages, could have prolonged survival times as the authors describe in the present article. Further basic and clinical research is necessary to improve our understanding of the biology of the different subtypes of DLBCL with plasmacytic differentiation in order to identify patients with a better prognosis and to develop newer therapeutic techniques.

## Consent

Written informed consent was obtained directly from 3 patients and from the parents of 1 patient for publication of this case report and any accompanying images.

## Competing interests

The authors declare that they have no competing interests.

## Authors' contributions

BB, RS, FH and LR referred the patients for this report. PQ and DM carried out pathology studies in Peru. JC performed the survival statistical analyses. JC and EW coordinated pathology studies in the U.S. BB, JC and EW prepared the manuscript. All authors read and approved the final manuscript.
